# Tobacco OPBP1 Enhances Salt Tolerance and Disease Resistance of Transgenic Rice

**DOI:** 10.3390/ijms9122601

**Published:** 2008-12-11

**Authors:** Xujun Chen, Zejian Guo

**Affiliations:** Key Laboratory of Plant Pathology, Ministry of Agriculture; Department of Plant Pathology, China Agricultural University, Beijing 100193, China. E-Mail: chenxj@cau.edu.cn

**Keywords:** AP2/EREBP, salt tolerance, disease resistance, transcription factor, transgenic rice

## Abstract

Osmotin promoter binding protein 1 (OPBP1), an AP2/ERF transcription factor of tobacco, has been demonstrated to function in disease resistance and salt tolerance in tobacco. To increase stress tolerant capability of rice, we generated rice plants with an *OPBP1* overexpressing construct. Salinity shock treatment with 250 mM NaCl indicated that most of the *OPBP1* transgenic plants can survive, whereas the control seedlings cannot. Similar recovery was found by using the seedlings grown in 200 mM NaCl for two weeks. The *OPBP1* transgenic and control plants were also studied for oxidative stress tolerance by treatment with paraquat, showing the transgenic lines were damaged less in comparison with the control plants. Further, the *OPBP1* overexpression lines exhibited enhanced resistance to infections of *Magnaporthe oryzae* and *Rhizoctonia solani* pathogens. Gene expressing analysis showed increase in mRNA accumulation of several stress related genes. These results suggest that expression of *OPBP1* gene increase the detoxification capability of rice.

## 1. Introduction

As sessile organisms, plants have developed adaptive strategies to cope with environmental stress by expressing specific genes and synthesizing a variety of proteins or compounds including transcription factors, late embryogenesis-abundant (LEA) proteins, transporters, antioxidants, proline, and sugars [1]. They are categorized either as effectors, which directly modulate or attenuate stress effects, or as regulators that are involved in stress perception, signal transduction, or regulation of effectors. LEA proteins have been considered to play roles in maintenance membrane structures, binding of water, and acting as molecular chaperones [2]. Sugar and other compatible solutes serve as osmoprotectants and stabilize biomolecules. For instance, accumulation of trehalose, a nonreducing disaccharide of glucose, in rice plants confers high tolerance levels to different abiotic stresses [3].

AP2/ERFs have been identified as a large gene family of plant transcription factors and contain a conserved basic 58 or 59-amino acid DNA-binding domain, defined as ERF domain [4, 5]. A comprehensive computational analysis identified 122 and 139 ERF family genes in *Arabidopsis* and rice, respectively [6]. ERF proteins have found to play important roles in plant adaptation to abiotic stress, such as drought, cold, salt oxidative stress and ABA response [7]. The barley ERF protein HvRAF enhances salt tolerance in *Arabidopsis* [8]. Three *Arabidopsis* ERFs, DREB1A, DREB2A, and CBF1, are induced in response to drought or cold stress [5, 9]. Further, ectopic expression of the genes such as *CBF1* or *DREB1A* has been shown to improve dehydration stress tolerance in *Arabidopsis* [10]. Dubouzet *et al*. [11] isolated five DREB homologs from rice, in which overexpression OsDREB1A increased tolerant to drought in *Arabidopsis*. In previous study, we have demonstrated that OPBP1, an ERF transcription factor isolated from tobacco is mediated in disease resistance and salt tolerance in tobacco [12]. In this report, we generated *OPBP1* overexpressing rice plants and found the transgenic progenies exhibited enhanced disease resistance against *Magnaporthe oryzae* and *Rhizoctonia solani*, as well as tolerance to salt stress.

## 2. Results

### 2.1. Expression of stress-inducible genes in OPBP1 transgenic plants

**Figure 1.**
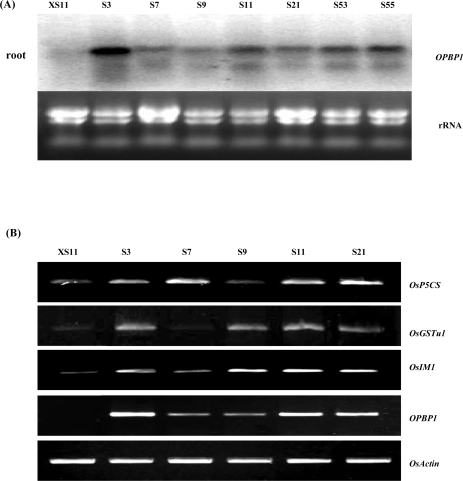
Constitutively expressing *OPBP1* and stress-inducible genes in transgenic rice plants. (A) Expression of *OPBP1* gene in *CoU::OPBP1* transgenic plants and in a control plant under normal condition was examined by Northern blot analysis using *OPBP1* cDNA as a probe. Numbers indicate independent lines of transgenic T_2_ plants. (B) Expression of *OPBP1*, *OsP5CS*, *OsGSTu1*, *OsIM1*, and *OsActin* were determined by RT-PCR. The mRNAs from control plants (XS11) and *CoU::OPBP1* lines were reverse transcribed and then used as templates for PCR. Actin gene was used as an internal standard. The amplification cycles were 25. Numbers indicate independent lines of transgenic T_2_ plants, and XS11 is the control.

Since OPBP1 was a regulator of disease resistance and salt tolerance in tobacco, we extended the study to examine a possible biological function of OPBP1 in rice. *OPBP1* gene was constructed under the control of a maize ubiquitin promoter (*Ubi:OPBP1*). Thirty independent lines were obtained by *Agrobacterium*-mediated transformation method. Expression of the transgene in some of T_2_ progenies was examined by northern blot analysis. As shown in Figure 1A, accumulation of *OPBP1* mRNA was increased in roots, although the level of accumulation varied between different lines.

To examine the induction of gene expression in *OPBP1* overexpressing plants, we chose several stress-inducible rice genes, such as delta-l-pyrroline-5-carboxylate synthetase (*OsP5CS*) [13], *OsGSTu1* [14] encoding for glutathione-S-transferase, and *OsIM1* [15], sharing homology with *AOX* members of *AtIM* and *PTOX.* Overall, expression of *OsGST*, *OsIM1* and *OsP5CS* genes was increased in the transgenic *OPBP1* plants in comparison with the control plants (Figure 1B).

### 2.2. Expression of OPBP1 enhances salt tolerance in rice

**Figure 2.**
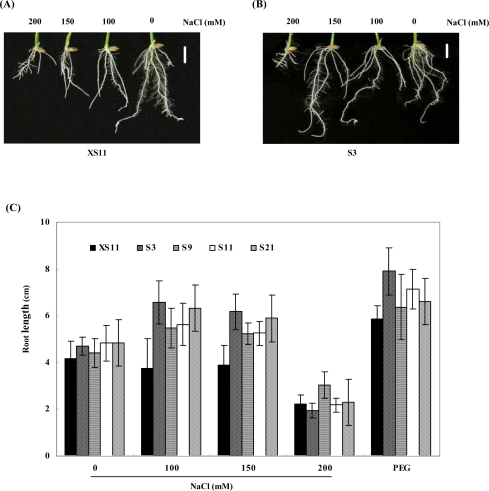
Effect of salt stress on rice seedlings of control and transgenic lines. The germinated seeds were planted onto 1/2MS with 200, 150, 100 mM or without NaCl, respectively. Photos were taken after 14 days of growth. (A) The root growth of control plant (XS11) under salt stress. (B) The root growth of overexpression line (S3) under salt stress. The bar indicates 2.0 cm. (C) The root length of each line under the same treatment. Values are means of at least 10 different plants in each line detected, and bars are standard errors. Numbers indicate independent lines of transgenic T_2_ plants, and XS11 is a control one.

To investigate the response to salinity, the transgenic rice seedlings of T_2_ progenies were grown for two weeks in 1/2 MS containing different concentrations of NaCl in the presence of hygromycin. The growth was suppressed with the increase of NaCl concentration both in the transgenic and the control plants. The heights in the presence of 200 mM NaCl were about one quarter of those without NaCl for both the control and transgenic lines (data not shown). Comparing the root growth between the transgenic and control plants, the length of roots in *OPBP1* overexpressing plants was even slightly increased in the presence of 100 or 150 mM NaCl, whereas the root length did not change significantly in the control plants under this condition (Figure 2). Increase of NaCl up to 200 mM, the root growth was inhibited in both the transgenic and control lines. The effects of salt on plants are considered to be caused by ionic toxicity and osmotic pressure [16]. To examine the osmotic effect, rice seedlings were treated with osmotic reagent PEG 6000 (12.1%, w/v), whose osmotic pressure was about that of 100 mM NaCl. At this concentration of PEG6000, the root growth was even stimulated slightly at the osmotic stress (Figure 2C). The results suggest that inhibition of rice growth by NaCl treatment is possibly due to the ionic toxicity.

**Figure 3.**
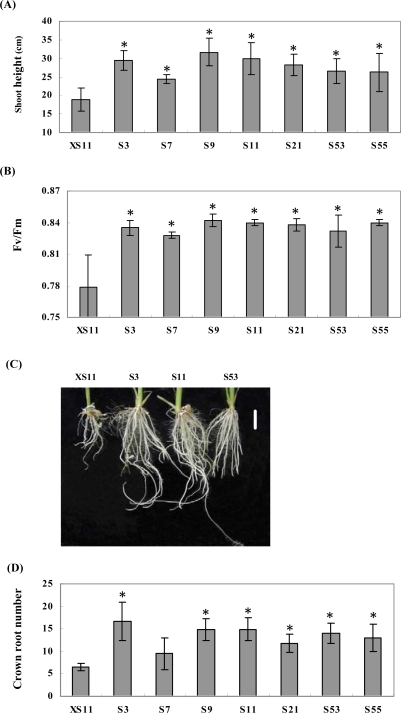
The recovery growth of rice plant after salt stress. The seedlings from the 1/2MS medium containing 200 mM NaCl were transferred to normal 1/2MS medium for another 14-day recovery. (A) The shoot height was measured. (B) The second leaf was taken to measure Fv/Fm. Values are means of at least 10 different plants in each line detected, and bars are standard errors. The values of the overexpressing lines differ significantly with the control’s (P<0.05) according to Fisher’s protected LSD means comparison procedures. (C) The root growth, the bar indicates 1.0 cm. (D) The crown root number was counted after 14-day recovery. Numbers indicate independent lines of transgenic T_2_ plants, and XS11 is a control. The values of the overexpressing lines except S7 differ significantly with the control’s (P<0.05) according to Fisher’s protected LSD means comparison procedures.

To examine the recovery capacity of salt-treated plants, the rice seedlings grown in 200 mM NaCl for two weeks were transferred to normal 1/2MS medium for recovery. After another two weeks, the control plants did not grow significantly, as revealed by measurement of the plant heights (Figure 3A).

In line with this, the Fv/Fm value of the control plants, a parameter often used as an indicator of the efficiency of primary photochemistry in photosystem II (PSII), decreased in comparing with the *OPBP1* expressing lines (Figure 3B). Importantly, the number of crown roots was more in the *Ubi:OPBP1* plants than the controls and the lengths of crown roots were longer (Figure 3C, D). Also, for most of the *OPBP1* transgenic plants, the average biomass of the seedlings were near double the weight of the controls (data not shown).

**Figure 4.**
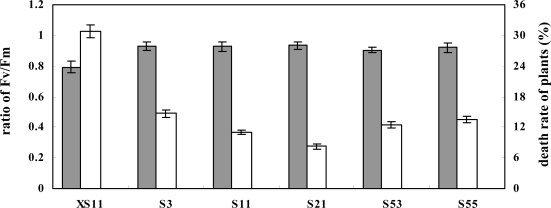
The recovery growth of rice plant after salt stress. The control plant (XS11) and transgenic plants were treated with 250 mM NaCl solution for 18 h, then they were allowed to grow under normal condition for recovery. The values of Fv/Fm were measured as described in Figure 3 and used as the ratio of day 4 to day one (the black column). The open column is the percentage of death plants.

Furthermore, the *OPBP1* transgenic and control plants were examined for their restorable capability after high salinity shock. Three-week-old rice seedlings were exposed to 250 mM NaCl for 18 h. With this treatment, about 37% of the youngest leaves of the control seedlings showed morphological wilting. Then, the treated seedlings were washed off and planted in soil for restoration. Within a seven-day-period of recovery, the control seedlings did not show obvious growth and about 31% of them died (Figure 4). On the other hand, the death of *OPBP1* overexpressing plants was significantly less compared with the control lines. The damage on the secondary leaves was more severe in the control lines than the *OPBP1* overexpressing plants, as indicated by the Fv/Fm values on day 4 after the treatment. New emergent leaves in the *Ubi:OPBP1* plants survived. The results suggest that overexpressing *OPBP1* increase the detoxification capability of rice.

### 2.3. Overexpression of OPBP1 increases tolerance to oxidative damage and resistance against rice fungal pathogens

**Figure 5.**
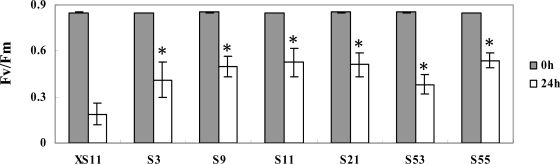
The effect of paraquat treatment on transgenic rice. The control plant (XS11) and transgenic plants were treated with 30 μM paraquat. Fv/Fm values were measured 24 h after the treatment. Values are means of 10 different pieces, and bars are standard errors. Numbers indicate independent lines of transgenic T_2_ plants. The values differ significantly (P<0.01) according to Fisher’s protected LSD means comparison procedures.

Since generation of reactive oxygen species and programmed cell death are mediated in salinity stress [17], the tolerance against oxidative stress of the *OPBP1* overexpressing and control plants was studied by treatment the plants with paraquat (Pq), which is a chemical producing superoxide anion and H_2_O_2_. Similarly, the Fv/Fm values were measured to evaluate the damage of the plants. As shown in Figure 5, decreases of Fv/Fm values were apparent in both the *OPBP1* overexpressing and the control plants 24 h after treatment with Pq. However, the *OPBP1* overexpressing plants showed less extent of decrease in Fv/Fm values comparing with the control plants, suggesting that overexpression of *OPBP1* stimulated the protective mechanism of rice plants against oxidative damage.

**Figure 6.**
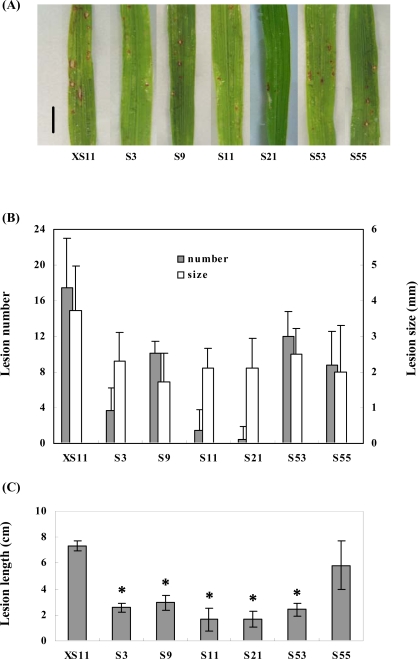
The resistance to *Magnaporthe oryzae* and *Rhizoctonia solani* of overexpression rice lines. (A) The phenotype after inoculated with *M. oryzae* 97-220E3 for 7 days. The bar indicates 3 mm. (B) Average lesion number and average lesion size (mm). Values are means of 20 different plants detected in each transgenic line, and bars are standard errors. The values differ significantly (P<0.05) according to Fisher’s protected LSD means comparison procedures. (C) Average lesion development length (cm) 13 days post inoculation. Values are means of 10 different plants detected in each transgenic line, and bars are standard errors. The values of the overexpressing lines except S55 differ significantly (P<0.05) according to Fisher’s protected LSD means comparison procedures.

Rice blast, caused by *Magnaporthe oryzae*, is a disastrous crop disease worldwide. To examine the effect of the transgene on disease resistance, the transgenic and control plants were inoculated with a virulent isolate of *M. oryzae* 97-220E3. Severity of disease development was evaluated by counting the number of lesions and measuring the size of lesions on infected leaves. The lesion numbers were reduced in the *OPBP1* overexpressing lines, especially lines 3, 11, and 21, comparing with the control plants (Figure 6A and 6B). Also, the lesions on the leaves of the transgenic plants were considerably smaller in size (Figure 6B).

Rice sheath blight is also a severe disease caused by *Rhizoctonia solani*. We inoculated the transgenic and control plants with an isolate of *R. solani* YN-7 and found that the lesion development was slower in the *OPBP1* overexpressing lines in comaprison with the control plants (Figure 6C).

## 3. Discussion

Genes induced by a stress are considered to play roles in responses to it. The early induced genes are required for stress perception, signal transduction or immediate protection, whereas those induced later are possibly involved in homeostasis and recovery. Overexpression of *OPBP1*, an early immediate gene, results in a high tolerance to salt stress and enhanced resistance against pathogen attacks in tobacco [12]. Also, several reports have demonstrated that overexpression of *ERF* genes enhances the ability of transgenic plants to withstand environmental cues. Ectopic expression of *CBF1/DREB1* genes activated cold responsive genes and enhanced cold and osmotic tolerance of non-acclimated *Arabidopsis* plants. DREB2, on the other hand, was found to be involved in drought-responsive gene expression [9, 10]. In this study, ectopic expression of *OPBP1* gene enhanced tolerance to salt stress as well as resistance against *M. oryzae* and *R. solani* pathogens, suggesting OPBP1 probably functions in a similar way in rice as in tobacco.

The damage cause by salt stress is often related to the production of oxidative stress. A number of reports have demonstrated that enhancement of the ROS scavenging systems in plants can provide protection from oxidative damage. For instance, overexpression of GST in tobacco showed substantial improvement of seedling growth under stressful condition including chilling and salinity [18]. This protective effect appears to scavenge products of lipid peroxidation generated as a result of enhanced ROS production. Overexpression of *OPBP1* gene increased the level of *OsGSTu* expression except of line S7 (Figure 1B). This low *OsGSTu* expression level in line S7 seems to coincide with its lesser restoration capability from salt-stress (Figure 3A, 3D). Furthermore, *OsIM* gene is induced by salt stress and has been estimated to decrease the content of ROS generated under salt-stress condition [15]. OsIM shares homology with AtIM from *Arabidopsis* and PTOX from tomato, which function as a plastid terminal oxidase for carotenoid synthesis in the phytogene de-saturation step. Carotenoids can prevent plants from oxidative stress by reacting with reactive oxygen species directly or indirectly [19]. Increase the level of *OsIM1* expression in *OPBP1* transgenic plants suggests again that the transgenic provides higher level of ROS scavenging capacity. In support of this, we observed a relative increase of paraquat tolerance in *OPBP1* overexpressing plants comparing with the controls (Figure 5).

One of the mechanisms for plant adaptation to water deficiency is the accumulation of low-molecular-weight organic compatible solutes such as sugars, some amino acids and quaternary ammonium compounds [20]. We examined the expression of some stress-related genes, such as *OsP5CS*, encoding a key enzyme for proline biosynthesis [13]. *OsP5CS* is induced by salt stress, cold stress, drought and the treatment of exogenous ABA [13]. The expression of *OsP5CS* and the accumulation of proline induced by high-salt treatment have been demonstrated to relate to the degree of salt tolerance in rice. In agreement, the increase in mRNA accumulation of *OsP5CS* in the transgenic plants suggest that overexpression of *OPBP1* may help avoid stress associated injuries and better recovery from stress. High salt stress disrupts homeostasis in water potential and ion distribution. To achieve salt tolerance, one of strategies is to help plants to re-establish both ionic and osmotic homeostasis in stressful environments. Increase osmotic stress by treatment with PEG increased root growths of transgenic and wild-type rice plants (Figure 3C), especially the increase in line S3 was significant different from the control plant coincident with the expression of *OPBP1* in line S3, which was the highest among the plants studied. The results suggest that the high level of expressing *OPBP1* in the transgenic plant probably lead more rapidly or strongly responsing to osmotic stress. The identification of molecular switches and regulatory genes would provide a better tool for crop improvement strategies.

## 4. Experimental Section

### 4.1. Construction of plasmids and rice transformation

To overexpress *OPBP1* gene in rice (*Oryza sativa* subsp. *japanica* var. Xiushui 11), the cDNA of *OPBP1* was digested with *Bam*H I/*Sac* I and inserted into pCoU linearized with the same enzymes. *OPBP1* gene was under the control of a maize ubiquitin promoter and terminated with an rbcs terminator in the generated plasmid (CoU-Ubi:*OPBP1*). Rice transformation was performed as described by Hiei *et al.* [21]. Hygromycin B-resistant plants were grown in the greenhouse and set seeds.

### 4.2. RNA isolation and northern blot

Total RNA from rice leaves was isolated using the Trizol Reagent Kit according to manufacturer’s instructions. An aliquot of 20μg total RNA was fractionated in a 1.0% denaturing agarose formaldehyde gel and subsequently transferred onto nylon membranes (Hybond-N+, Amersham). Hybridizations were performed with ^32^P-labeled *OPBP1* cDNA fragment as described previously [12].

### 4.3. Chemical treatments

The rice seeds of T_2_ progenies were surface sterilized with 70% ethanol for 5 min and with 2.5% NaClO for 30 min. They were washed thoroughly, soaked in distilled water for 48 h, and incubated for 24 h at 30 °C in the dark conditions. The germinated seeds were sown in 300 mL plastic cups with dark cloth outside. The seedlings were grown on the medium containing 1/2 MS salts without sucrose and organic ingredients, in the presence of 0, 100, 150, and 200 mM NaCl, respectively. For osmotic treatment, we used a 12.1% PEG6000 solution, which had a similar osmotic potential (–0.58 Mpa) as 100 mM NaCl solution. After 14 days growth at 25 °C with a day/night cycles of 16/8 h, the heights and root length of the seedlings were measured.

To examine the recovery capability, the seedlings grew in 200 mM NaCl for two weeks were transfered to a new 1/2 MS medium in the absence of NaCl for 14 days more growth. Then, the seedlings were harvested for measurement of parameters. In order to investigate the seedlings tolerance to salt shock, the germinated seeds of the T_2_ and the control lines were sown in pots filled with a mixture of soil and vermiculite (1:1, v/v) and grown in the field under a 14/10 h (light/dark) photoperiod at 27 °C for 3 weeks with daily watering. The seedlings were treated or not with 250 mM NaCl for 18 h. After this treatment, the seedlings were washed with water and allowed to grow under normal condition for another 35 days. The values of Fv/Fm of the secondary leaves were determined at the planned time. The dry weights were measured after placing fresh seedlings in an 80 °C oven for 72 hours.

For paraquat treatment, leaf blade pieces about 3 cm in length each from the secondary leaves of 3-week-old seedlings were floated on a 30μM paraquat solution. Equivalent leaf blade sections were incubated on sterile water without paraquat treatments as a control. The leaf blade sections were incubated for 24 h at 25 °C under continuous illumination (200 W/m^2^/s) until sampling.

### 4.4. Determination of chlorophyll fluorescence

Measurement of chlorophyll fluorescence was performed with a fluorometer. Fluorescence signals were from the second leaf of each rice plant, which had been dark-adapted for 15 min, were measured at the indicated times. The ratio of Fv to Fm (Fv/Fm) representing the activity of photosystem II was used to assess the functional damage to the plants [22].

### 4.5. Pathogen inoculation

A virulent (97-220E3) isolate of the blast fungus *Magnaporthe oryzae* was used in this study. Three-week-old seedlings of control and *OPBP1* lines were inoculated with 97-220E3 at a concentration of 3×10^5^ spores per ml, following the procedure described by Sallaud *et al.* [23]. After incubation in a dark chamber (at 22 °C and 95% relative humidity) for 24 h, plants were moved to a growth chamber and maintained at 28 °C with 16 h of light and 8 h of dark cycles. Disease symptoms were evaluated and photographed. Number of lesions per leaf was counted as the size of the lesions was measured. The experiment was repeated three times in rice seedlings with similar results.

The isolate of *Rhizoctonia solani* (YN-7) was used in this study. Three-week-old seedlings of control and *OPBP1* lines were inoculated with YN-7, following the procedure described by Jia *et al.* [24]. The greenhouse temperate averaged 28 °C, and relative humidity was maintained within 70% to 80%. Lesion development was monitored starting 3 days post inoculation. The length of the lesion was measured. Sheath blight micro-chamber inoculations were repeated twice.

### 4.6. RT-PCR analysis

To remove possible DNA contamination, the total RNA was treated with DNase, then was heated at 70 °C for 10 min to inactivate the DNase, then followed by phenol-chloroform extraction and ethanol precipitation. The first strand cDNAs were synthesized by using the AMV reverse transcriptase system in 20 μL reactions, including 1 μg purified total RNA. After synthesis, the cDNA was diluted and used as template for PCR amplifications in 50 μL standard reactions. For RT-PCR, actin was used as internal constitutive control. The PCR products were separated on 1.6% w/v agarose gels, stained with ethidium bromide, and photographic images were obtained. The primers used in the study were as followings: rice actin (GenBank accession number: AB047313), OsACTIN-F: 5’-ggaactggtatggtcaaggc-3’ and OsACTIN-R: 5’-agtctcatggatacccgcag-3’; *OPBP1* (GenBank accession number: U81157)*,* OBP1BI: 5’-acgaggaaaaacaaaatggattct-3’ and OBP1R: 5’-atggaaacaaagagatggaattcccctat-3’; *OsP5CS* (GenBank accession number: D49714), OsP5CS-F: 5’-tggatgtctcgtcatctcaact-3’ and OsP5CS-R: 5’-aagccaagacagcagccttcac-3’; *OsGSTu1* (GenBank accession number: AF050102), OsGSTu1-F: 5’-aagctatagccatggcggaggag-3’ and OsGSTu1-R: 5’-tgcaacttcgcatccaccgatctac-3’; *OsIM1* (GenBank accession number: AF085174), OsIM1-F: 5’-gggagaaggagcagaccga-3’ and OsIM1-R: 5’-gccacccaattcttccatg-3’. Transcript abundance of *OsActin* in leaves was 25cycles of PCR amplification.

## 5. Conclusions

*OPBP1* gene, an AP2/ERF transcription factor of tobacco, seems to be an important determinant of biotic and abiotic stress response in plants. Transgenic rice plants with overexpressed *OPBP1* gene accumulated higher levels of *OsP5CS*, *OsGSTu1* and *OsIM1*, which are reported to be involved in the signaling pathways of salt or oxidative stress tolerant. *OPBP1* overexpression lines exhibited enhanced tolerance to salt and oxidative stress, as well as to infection of *Magnaporthe oryzae* and *Rhizoctonia solani* pathogens. These results suggest that expression of *OPBP1* gene increase the detoxification capability of rice.
